# ACTH Action on Messenger RNA Stability Mechanisms

**DOI:** 10.3389/fendo.2017.00003

**Published:** 2017-01-20

**Authors:** Agnès Desroches-Castan, Jean-Jacques Feige, Nadia Cherradi

**Affiliations:** ^1^Institut National de la Santé et de la Recherche Médicale, INSERM U1036, Grenoble, France; ^2^Commissariat à l’Energie Atomique et aux Energies Alternatives, Institut de Biosciences et Biotechnologies de Grenoble, Laboratoire Biologie du Cancer et de l’Infection, Grenoble, France; ^3^Université Grenoble Alpes, Unité Mixte de Recherche-S1036, Grenoble, France

**Keywords:** ACTH, AU-rich elements, RNA-binding proteins, TIS11b/BRF1, HuR, tristetraprolin, mRNA stability

## Abstract

The regulation of mRNA stability has emerged as a critical control step in dynamic gene expression. This process occurs in response to modifications of the cellular environment, including hormonal variations, and regulates the expression of subsets of proteins whose levels need to be rapidly adjusted. Modulation of messenger RNA stability is usually mediated by stabilizing or destabilizing RNA-binding proteins (RNA-BP) that bind to the 3′-untranslated region regulatory motifs, such as AU-rich elements (AREs). Destabilizing ARE-binding proteins enhance the decay of their target transcripts by recruiting the mRNA decay machineries. Failure of such mechanisms, in particular misexpression of RNA-BP, has been linked to several human diseases. In the adrenal cortex, the expression and activity of mRNA stability regulatory proteins are still understudied. However, ACTH- or cAMP-elicited changes in the expression/phosphorylation status of the major mRNA-destabilizing protein TIS11b/BRF1 or in the subcellular localization of the stabilizing protein Human antigen R have been reported. They suggest that this level of regulation of gene expression is also important in endocrinology.

## Introduction

Transcriptional regulation of the cellular responses to hormones has been the primary focus of many endocrinological research studies during the past decades. Although the transcriptional mechanisms that regulate the production of specific mRNAs are undoubtedly important, it has become increasingly evident that processes regulating the stability of mRNAs also represent critical steps in the control of dynamic gene expression. In particular, acute changes in gene expression are now recognized to be controlled by RNA-binding proteins (RNA-BP) and microRNAs through their binding to target transcripts and their positive or negative regulation of mRNA turnover. Because the ability to respond to rapid changes in ACTH levels is essential for maintaining steroid hormone homeostasis, posttranscriptional mechanisms are expected to be involved in ACTH action. The pleiotropic effects exerted by ACTH on adrenocortical cell functions are regulated through a multiplicity of mechanisms. Through binding to its adenylate-cyclase-coupled receptor MC2R, ACTH stimulates the release of cAMP and the activation of the cAMP-dependent protein kinase A (PKA), which in turn phosphorylates and regulates a number of specific substrates including the steroidogenic acute regulatory protein (StAR) (1) and the cAMP response element-binding protein (CREB) (2). ACTH also strongly regulates the transcription of a number of genes involved in the steroidogenic response including those encoding several steroidogenic enzymes (3), components of the extracellular matrix (4) and many others. Another less characterized level of regulation through which ACTH exerts its actions is the control of mRNA stability through the activity of specific proteins that bind the 3′-untranslated region (3′-UTR) of target mRNAs. We were first to observe that the increase in vascular endothelial growth factor-A (VEGF-A) mRNA induced by ACTH in primary adrenocortical fasciculata cells did not result from increased transcription (5) but from stabilization of its mRNA (6). Here, we will present the proteins that mediate the regulation of short-lived mRNA stability/degradation and focus on those which are regulated by ACTH in adrenocortical cells.

## mRNA Stability Mechanisms: An Interplay Between *cis*-acting Elements and *trans*-acting Factors

**Figure 1:**
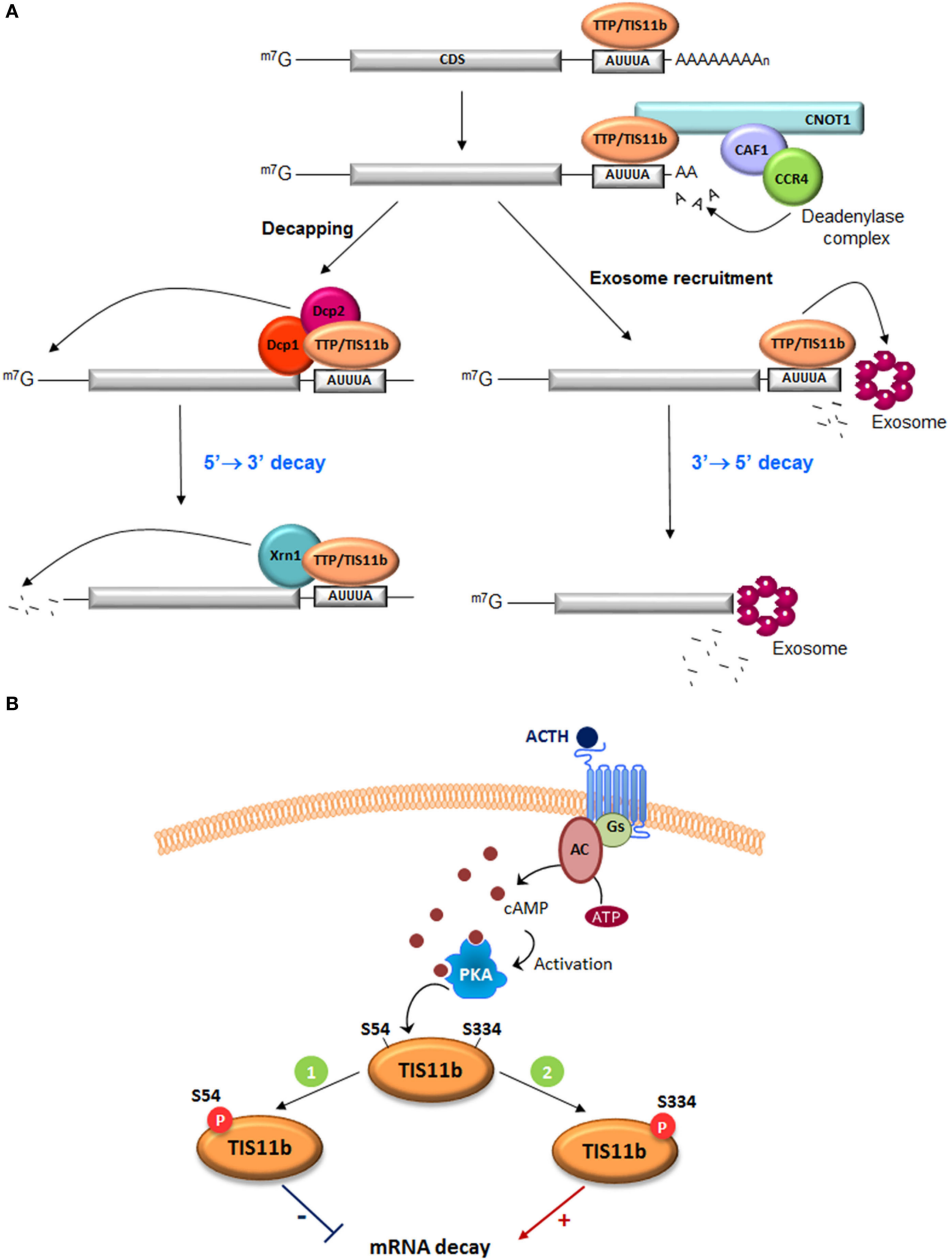
**Function of tristetraprolin (TTP) and TIS11b in ARE-mediated mRNA decay**. **(A)** TTP and TIS11b bind to AU-rich elements in the 3′-UTR of target mRNAs and recruit the deadenylase complex directly (CCR4–CAF–NOT1 complex) to trigger mRNA deadenylation. Deadenylated transcripts are degraded through TTP- or TIS11b-mediated recruitment of the exosome, a multiprotein complex that promotes the 3′ to 5′ mRNA decay. Alternatively, deadenylation can be followed by mRNA decapping by the decapping enzymes Dcp1/Dcp2 and the 5′ to 3′ mRNA degradation by the Xrn1 exonuclease. CDS, coding sequence. **(B)** Two putative protein kinase A (PKA) phosphorylation sites, S54 and S334, were identified in TIS11b protein sequence with important roles in protein activity and stability. ACTH stimulation increases intracellular cAMP levels through the action of the G protein Gs and the adenylyl cyclase. This leads to activation of PKA (1). Phosphorylation of TIS11b at S54 by PKA inhibits protein activity. TIS11b-phospho-S54 is sequestered in the cytoplasm due to enhanced interaction with 14-3-3 proteins. This mechanism would promote vascular endothelial growth factor (VEGF) mRNA induction (2). To turn down VEGF production, phosphorylation of TIS11b at S334 by PKA increases protein stability and activity. Dephosphorylation of both serines presumably by the phosphatase PP2A leads to degradation of TIS11b *via* the proteasome.

The steady-state level of any mRNA in an eukaryotic cell results from the balance between its synthesis through gene transcription and its degradation through the mRNA decay machinery. Regulation of mRNA stability implies both *cis* elements mainly located in the 3′-UTR of mRNAs and *trans*-acting factors. These latter factors comprise a number of RNA-BP that specifically bind distinct *cis* elements, form multimolecular scaffolds that favor or prevent the subsequent recruitment of the mRNA deadenylation and mRNA degradation machineries (Figure 1A) (7). Eukaryotic mRNAs are protected at both extremities by a 7-methyl guanosine cap at their 5′-end that confers resistance to 5′ to 3′ exonucleases and by a poly(A)-tail recruiting the poly(A)-binding protein (PABP) at their 3′-end that confers resistance to 3′ to 5′ exonuclease attack by the exosome complex (8). The most frequently distributed and best-studied *cis* elements are the AU-rich elements (AREs) that are often arranged in mRNA 3′-UTRs as repeated AUUUA pentameric sequences that eventually overlap (9). More than 6,000 human ARE-containing mRNAs have been listed in the last upgrade of the ARED 3.0 database (http://brp.kfshrc.edu.sa/ARED/) (10). Many of them have a short half-life, rendering this regulatory process highly effective to rapidly turn down a cellular function.

## AREs Binding Proteins

Among the more than 20 ARE-binding proteins (ARE-BP) identified so far, some are destabilizing the target mRNA by recruiting deadenylases and exonucleases and some are stabilizing them by protecting them from these degradation enzymes. The competition between both types of proteins for a given ARE will ultimately determine the fate of the target mRNA. The best characterized stabilizing ARE-BP is human antigen R (HuR), which is a member of the ELAV (homologs of the *Drosophila* proteins *embryonic lethal abnormal vision*) family (11). HuR is ubiquitously expressed and is predominantly localized in the nucleus of non-stimulated cells where it forms messenger ribonucleoprotein complexes that are assembled during splicing of primary transcripts, prior to transport of mature mRNAs to the cytoplasm (12). Upon cell activation by various stimuli, HuR undergoes CRM1-dependent nuclear-cytoplasmic shuttling, directed by localization signals (13). Binding of HuR to ARE may play important roles in controlling the processing, splicing, and polyadenylation of the nuclear transcript, together with the nuclear export and stabilization/translation in the cytoplasm. The exact mechanism by which HuR stabilizes target mRNAs is still unclear, but HuR has been reported in many cell types to prevent the degradation of target mRNAs by competing with destabilizing proteins and thereby preventing their recruitment of the exosome machinery (6, 14). A larger number of destabilizing proteins has been described. Among them, the tristetraprolin (TTP) family, which comprises three distinct members in mammals (TTP/TIS11/ZFP36, TIS11b/BRF1/ZFP36-L1, and TIS11d/BRF2/ZFP36-L2) is one of the best characterized. Although they all bind to similar synthetic sequences *in vitro*, members of the TTP family present specific sites of action and preferential targets *in vivo*, as demonstrated by the distinct phenotypes of the mice that have been genetically invalidated for each of these genes. In TTP-KO mice, the TNFα mRNA is significantly stabilized in macrophages, resulting in a fatal postnatal inflammatory syndrome (15). Deletion of TIS11b/BRF1 gene in mice results in embryonic lethality due to abnormal placentation and major vascular defects (16, 17). This is mainly caused by the failure of TIS11b to repress the expression of VEGF at the end of the developmental angiogenic process. The complete invalidation of the TIS11d gene causes postnatal lethality due to defective definitive hematopoiesis (18). Structural studies have shown that the tandem zing finger repeats of TTP family proteins bind to the 5′-UUAUUUA(U/A)(U/A)-3′ recognition motif while the C-terminal and N-terminal domains of the proteins participate in the recruitment of the mRNA deadenylation and mRNA degradation enzymes (19, 20). Other destabilizing ARE-BP include KH-splicing regulatory protein (KSRP) and the AU-rich RNA-binding factor 1 (AUF1/HnRNP D).

## ACTH Action on mRNA Decay Mechanisms: From Transcription to Phosphorylation of ARE-BP

TIS11b, also named BRF1 or ZFP36-L1, was identified in the adrenal cortex through a differential display RT-PCR analysis of ACTH-stimulated versus non-stimulated adrenocortical cells (21). HuR and alternatively spliced isoforms of AUF1/hnRNPD are also expressed in adrenal cells (6, 22). Recently, it was established that ACTH induced *zfp36-L1* gene transcription in bovine adrenocortical cells (BAC) through phosphorylation of CREB transcription factor and CREB-mediated activation of TIS11b promoter (23). A highly conserved cAMP response element (CRE) was found at -402 to -394 relative to the transcription start site (TSS) of human *zfp36-L1* gene. TIS11b mRNA is also rapidly stimulated by 8-bromo-cAMP in murine adrenocortical Y-1 cells or Leydig MA-10 cells (22), suggesting that the protein is a key regulator of endocrine tissue biology. Interestingly, no classical CRE was identified within the −2,000 bp upstream of the TSS of the two other family members TTP and TIS11d. In agreement with this observation, TTP or TIS11d mRNA levels were not changed upon ACTH challenge, pointing at a specific induction of TIS11b by the hormone in the adrenal cortex (23).

Two major target transcripts of TIS11b have been identified in the adrenal cortex. The first one is the message encoding the angiogenic cytokine VEGF (24). Knowing that VEGF mRNA was rapidly but transiently induced by ACTH in fasciculata cells through a transcription-independent mechanism, the contribution of TIS11b and HuR to this transient induction was investigated. Using HuR- and TIS11b-specific siRNAs, it was established that (i) ACTH induced nucleo-cytoplasmic translocation of HuR to trigger VEGF mRNA stabilization in the cytoplasm and (ii) TIS11b, which is induced later by ACTH, participates in the downregulation of VEGF mRNA levels. A short 75-bp long sequence in VEGF mRNA 3′-UTR was shown to bind TIS11b through two adjacent UUAUUUAAU and AUUUA motifs. The second identified target of TIS11b in endocrine cells is the StAR mRNA whose PKA-stimulated transcription paralleled TIS11b induction (22). *StAR* mediates intramitochondrial cholesterol transport in most steroidogenic tissues in response to hormonal changes (1). Cyclic AMP stimulates two major StAR transcripts of 3.5 and 1.6 kb, which arise from differential use of polyadenylation signals and therefore differ only in their 3′-UTR (lengths 0.7 and 2.8 kb, respectively). In mouse MA-10 and Y-1 cells, 8-bromo-cAMP stimulates StAR 3.5-kb mRNA as the predominant form. In BAC, similar long and short forms appear equally (21), whereas in human adrenocortical carcinoma cells H295R, di-butyryl-cAMP selectively stimulates the short form of StAR mRNA (25). Following translation, the 3.5-kb StAR message is preferentially degraded after removal of the stimulus through the action of TIS11b binding to UAUUUAUU repeats in the extended 3′-UTR. This attenuation process provides a rapid mechanism to inactivate StAR when hormonal stimulation ceases.

ARE-binding proteins are also distal targets of several signaling pathways. TTP and TIS11b are phosphorylated by a variety of protein kinases. However, the impact of these phosphorylations on their mRNA-destabilizing activity is still a matter of debate, with some studies indicating that phosphorylations of TTP decrease its affinity for ARE-rich mRNAs (26) and other studies reporting that phosphorylations by ERK, p38 MAP-Kinase/MK2, or JNK kinase do not impact this affinity (27). Similarly, phosphorylation of TIS11b by PKB/Akt or MK2 at conserved serine residues did not affect its ability to bind AREs but nevertheless inhibited its ability to promote ARE-mRNA degradation (28, 29). The current accepted model suggests that protein-kinase activation leads to phosphorylation of TTP or TIS11b, favors their sequestration by 14.3.3 proteins, and thereby reduces their mRNA-destabilizing action. Upon signal extinction, dephosphorylation of TTP and TIS11b occurs probably *via* protein phosphatase PP2A and allows the recruitment of the mRNA decay ribonucleases. A new ACTH-regulated phosphorylation site recently identified in TIS11b seems, however, to mediate a different biological response. ACTH-mediated activation of PKA was reported to induce the phosphorylation of TIS11b on two serine residues, S54 and S334 (23) (Figure 1B). Analysis of phospho-dead (S54A, S334A) and phospho-mimick (S54D, S334D) TIS11b mutants revealed that S54 regulates the binding of TIS11b to 14.3.3 protein but that S334 does not play a significant role in this interaction. In contrast, the C-terminal S334 phospho-site appears to be involved in the interaction with the CCR4–NOT1 deadenylation complex. TIS11b S334D phospho-mimick mutants presented a reduced association with CNOT1, a core subunit of the CCR4–NOT deadenylase complex, but however displayed an enhanced interaction with the decapping enzyme Dcp1a. Unexpectedly, this interaction was associated with an increased mRNA-destabilizing activity of TIS11b S334D as compared to the dephospho-form of TIS11b (23). These observations suggest that combinatorial phosphorylations of TIS11b on specific residues do not systematically abrogate their mRNA-destabilizing capability but rather fine-tune their interactions with the mRNA decay machineries.

## Expression of TTP Family Members in Adrenocortical Tumors

**Figure 2:**
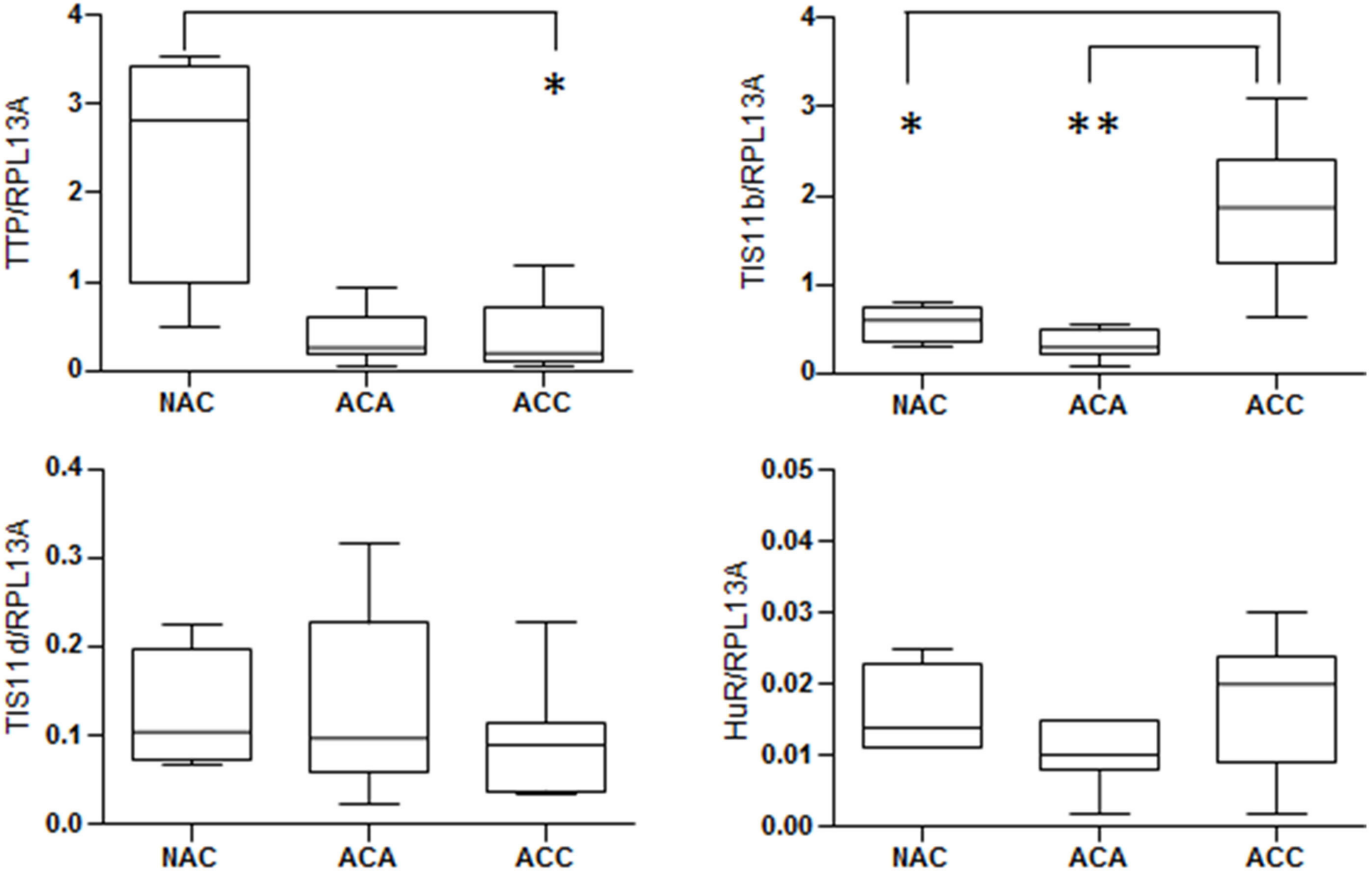
**Expression of mRNA stability regulators in human adrenocortical tumors**. Relative expression of mRNA stability factors in adrenocortical tumor samples from the French COMETE (COrtico et MEdullosurrénales, Tumeurs Endocrines) Network. TTP family members (TTP, TIS11b, and TIS11d) and HuR were quantified by reverse transcription-quantitative PCR in 4 normal adrenal cortex, 11 cortisol-producing adrenocortical adenomas, and 15 adrenocortical carcinomas. RPL13A was used as housekeeping gene for normalization. The graphs show median with interquartile range. All data were analyzed using the GraphPad Prism Software and were considered as statistically different when *p* < 0.05 (*) and *p* < 0.01 (**).

Overexpression of ARE-containing transcripts encoding factors promoting growth, inflammation, angiogenesis, and invasion has been observed in carcinogenesis (30). These aberrant expressions results from dysfunctional ARE-mediated posttranscriptional control, which seems to be mainly due to deregulations in ARE-binding proteins rather than to ARE mutations. Downregulation of TTP expression has been found in a variety of human malignancies including breast, colon, prostate, and lung cancers (31–33). The loss of TTP expression seems to be an early event during tumorigenesis. Nevertheless, apart from single-nucleotide polymorphisms associated with decreased translation efficiency, the mechanisms leading to TTP suppression in cancer remain obscure. Analysis of the mRNA expression levels of TTP family members (TTP, TIS11b, and TIS11d) and HuR in human adrenocortical tumors revealed that TTP mRNA was dramatically decreased in adrenocortical adenoma (ACA) and adrenocortical carcinomas (ACC) as compared to normal adrenal cortex (NAC) (Figure 2). By contrast, TIS11b mRNA is highly expressed in ACC as compared to ACA and NAC while the expression of the third member of the family TIS11d was similar in all the tissues examined. No significant difference in HuR expression was found between normal cortex and adrenocortical tumors. Remarkably, the expression patterns of TTP and TIS11b are symmetrically opposite in normal adrenal cortex and malignant tumors. The relevance of these variations to human physiology and the pathology of adrenocortical cancer remains to be determined. In addition, these results will require validation by measurement of TTP and TIS11b protein levels.

## Closing Remarks and Perspectives

Transcriptional regulation has been considered the primary control point of protein production in eukaryotic cells. However, there is growing evidence of pivotal posttranscriptional regulation for many genes, including those involved in differentiated functions of the adrenal cortex such as the *StAR* gene. This has prompted extensive investigations to elucidate the mechanisms controlling RNA processing, mRNA nuclear export and localization, mRNA stability, and turnover, in addition to translational rates and posttranslational events. The regulation of mRNA stability has emerged as a critical control step in determining the cellular mRNA level, which is regulated through specific RNA sequence elements–protein interactions. In this context, study of the hormonal control of mRNA stability regulatory proteins and their activity in adrenal cortex function is just beginning. Considering that acute ACTH treatment affects a large number of transcripts, it seems very likely that mRNA stability regulations might play an important role in these transient gene expressions. These mechanisms are expected to also operate in response to other cAMP-mobilizing hormones in their respective target organs. For example, parathyroid hormone has been shown to decrease sodium/hydrogen exchanger 3 mRNA stability through the action of the destabilizing protein KSRP in kidney epithelial cells (34). Importantly, few mRNA stability regulatory factors have been identified so far that appear to control a large pool of target mRNAs. This suggests that a slight alteration in the control mechanism may generate large-scale effects that could contribute to the development of complex disorders, including adrenal diseases. Efforts in studying mRNA stability regulators in adrenal cortex and their hormonal regulations should be made in order to better understand their potential contribution to adrenocortical pathologies and possibly discover potential biomarkers and therapeutic targets.

## Author Contributions

All authors listed have contributed to the work.

## Conflict of Interest Statement

The authors declare that the research was conducted in the absence of any commercial or financial relationships that could be construed as a potential conflict of interest.

## Abbreviations

TIS11b, TPA-inducible sequence 11b; BRF1, butyrate response factor 1; ZFP36, zinc finger protein; TTP, tristetraprolin; CCR4, carbon catabolite repressor protein 4; CNOT1, CCR4-NOT transcription complex subunit 1; Dcp1a, decapping enzyme 1a.
